# Safety and effectiveness of clofarabine in Japanese patients with relapsed/refractory acute lymphoblastic leukaemia: a post-marketing surveillance study

**DOI:** 10.1093/jjco/hyae047

**Published:** 2024-04-20

**Authors:** Hirotaka Kazama, Satoshi Nishina, Takeshi Seto

**Affiliations:** Specialty Care Oncology Medical, Sanofi, Tokyo, Japan; Medical Affairs, Post-Authorization Regulatory Studies, Sanofi, Tokyo, Japan; Medical Affairs, Post-Authorization Regulatory Studies, Sanofi, Tokyo, Japan

**Keywords:** acute lymphoblastic leukaemia, clofarabine, post-marketing surveillance

## Abstract

**Objective:**

Clofarabine is used to treat acute lymphoblastic leukaemia, but evidence of its safety and effectiveness in Japanese patients is limited. We evaluated the safety and effectiveness of clofarabine in patients with relapsed/refractory acute lymphoblastic leukaemia in real-world clinical practice in Japan.

**Methods:**

An observational, multicenter, post-marketing, all-case surveillance was conducted for safety. Effectiveness analyses were conducted in patients aged ≤21 years and those treated with clofarabine monotherapy and combination therapy (clofarabine plus etoposide and cyclophosphamide).

**Results:**

In the all-case survey, 260 of 264 registered patients were eligible for safety analysis. Among the 225 patients eligible for effectiveness analysis, 139 were aged ≤21 years. For monotherapy and combination therapy, 20/31 and 34/88 patients were eligible, respectively. In the all-case survey, the median age was 16.0 years, and 47.7% of patients were <15 years old. Adverse drug reaction incidence was 83.5% and the most common were hematologic toxicities. The best overall response rates in the population aged ≤21 years were complete remission, 29.7%; complete remission without platelet recovery, 7.3% and partial remission, 10.9%. The rest (52.2%) were classified as ineffective. The sum of complete remission, complete remission without platelet recovery and partial remission rates (effectiveness rate) was 47.8% (66/138 patients). The effectiveness rates in the monotherapy and combination therapy surveys were 10.0% (2/20 patients) and 58.8% (20/34 patients), respectively.

**Conclusions:**

These post-marketing surveys provide real-world evidence of the safety and effectiveness of clofarabine regimens, including monotherapy and combination therapy in Japanese patients with relapsed/refractory acute lymphoblastic leukaemia. The safety and effectiveness profiles were comparable with those of previous prospective studies.

## Teaser

These studies provide real-world evidence of the safety and effectiveness of clofarabine administered as monotherapy and in combination with etoposide and cyclophosphamide in Japanese patients with relapsed/refractory acute lymphoblastic leukaemia.

## Introduction

Acute lymphoblastic leukaemia (ALL) is a malignant haematological disease that affects both children and adults, with most cases occurring in healthy individuals (1). It has been estimated that despite chemotherapy, 10–15% of paediatric patients with ALL and 40–50% of adult patients with ALL experience relapse (2–4). Disease relapse is the leading cause of death among patients with ALL; after relapse, the median survival ranges from 3 to 8.4 months (4–6). Although overall survival has improved in the last decade with advances in treatment, outcomes of patients with refractory or relapsed ALL remain poor after conventional chemotherapy.

Clofarabine is a second-generation purine nucleoside analogue. Its cytotoxic effect depends on intracellular phosphorylation to the triphosphate metabolite (7). Clofarabine exerts its antitumour effect by reducing the synthesis of deoxynucleotide triphosphates, inhibiting DNA polymerase, increasing incorporation of clofarabine triphosphate into DNA and eventually inducing mitochondria cytochrome c release through DNA damage, which induces apoptosis (8).

Previous Phase 1 and 2 studies have evaluated clofarabine administered as monotherapy or combined with etoposide and cyclophosphamide in patients with ALL (9–16). Most of these studies showed encouraging activity of clofarabine in patients with acute leukaemia and refractory or relapsed ALL. However, a Phase 1 study of clofarabine monotherapy in Japanese paediatric patients with ALL showed that there were no responders (patients evaluated as having complete remission [CR], CR without platelet recovery [CRp] or partial remission [PR]) because only seven paediatric patients were enrolled for tolerability in that clinical trial (13). In addition, the safety profile of clofarabine has yet to be fully investigated in Japanese patients.

The present report summarizes a post-marketing surveillance conducted to evaluate the safety and effectiveness of clofarabine in real-world clinical practice in Japan. The main study (all-case survey) aimed to confirm the real-world safety and effectiveness of clofarabine in patients with relapsed/refractory ALL and identify the following: unexpected adverse drug reactions (ADRs), the occurrence of ADRs in real-world clinical practice and the occurrence of adverse events (AEs) of special interest. The additional studies aimed to evaluate the effectiveness of clofarabine monotherapy and clofarabine in combination with etoposide and cyclophosphamide in real-world clinical practice in Japanese patients with relapsed/refractory ALL.

## Patients and methods

### Study design, treatment and patients

Three observational, multicenter, post-marketing surveillances were conducted from 18 September 2013 to 19 September 2018 (all-case survey), 26 June 2014 to 8 April 2018 (monotherapy survey) and 19 May 2014 to 9 March 2020 (combination therapy survey) in Japan. Of the patients enrolled in the all-case survey, those administered clofarabine monotherapy or combined with etoposide and cyclophosphamide were simultaneously enrolled in each outcome surveillance. As the number of patients who received the combination regimen was insufficient when the registration of the all-case survey was completed, only the combination regimen cases continued to be registered after the completion of the all-case survey.

Clofarabine 52 mg/m^2^ (body surface area) was administered by intravenous infusion over 2 h daily for five consecutive days and then discontinued for at least 9 days (Cycle 1) until the next cycle (for a maximum of six cycles). Among patients who received combination therapy, clofarabine was administered at a dose of 52 mg/m^2^, and etoposide and cyclophosphamide were administered at a dose of 150 and 400 mg/m^2^, respectively. The intravenous infusion was administered over at least 2 h daily for five consecutive days, then discontinued for at least 9 days (Cycle 1) until the next cycle. The dose of clofarabine could be decreased depending on the patient’s condition.

The observation period was defined as the period between the start of the study drug to the end of the final cycle (at least 9 days from the end of the 5-day treatment period). The observation period was set to a maximum of 6 cycles. Patient data were collected from case report forms (CRFs).

The institutional review board approved the protocol at each study site. The study was conducted in accordance with the Declaration of Helsinki and adhered to the guidelines for Good Post-marketing Study Practice in Japan. Informed consent was exempted under Good Post-marketing Study Practice. Agreements were made with the study sites to publish anonymized data.

### Safety assessments

ADRs, serious ADRs, AEs of special interest (including hematotoxicity, infection, renal disorders, hepatobiliary disorders, capillary leak syndrome and systemic inflammatory response syndrome [SIRS], tumour lysis syndrome and cardiovascular events) and abnormal changes in laboratory parameters were evaluated. AEs were coded using the Medical Dictionary for Regulatory Activities, version 23.1, and graded according to the Common Terminology Criteria for Adverse Events, version 4.0.

### Effectiveness assessments

Response to treatment was evaluated by the best overall response (CR, CRp or PR), the remission rate was evaluated by CR + CRp and the effectiveness rate was evaluated by CR + CRp + PR. The criteria for CR were defined as meeting all of the following: no leukaemic cells in peripheral blood and no extramedullary infiltration, <5% of leukaemia cells in bone marrow and platelet count and neutrophil count in peripheral blood recovered to ≥100 000 and ≥1000/mm^3^, respectively. The criteria for CRp were the same as for CR criteria except platelet count recovery (≥100 000/mm^3^). The definition of PR was: no leukaemic cells found in peripheral blood and leukaemic cells in bone marrow >5% and <25% with normal blood cell precursors observed (or if <5% of leukaemic cells in bone marrow, but not meeting the criteria for CR and CRp). Ineffective was defined as not meeting the CR, CRp or PR criteria.

The effectiveness analysis was performed for patients with relapsed/refractory ALL aged ≤21 years because the target population of clinical trials in Japan and overseas at the New Drug application submission in Japan was ≤21 years of age. In addition, the effectiveness was also summarized for patients aged ≥22 years.

### Statistical methods

The minimum number of cases required to detect AEs occurring at a frequency of 3% with a probability of ≥95% was 100 cases. Thus, 120 patients were set as the target sample size. Baseline demographic and clinical characteristics were evaluated in the safety analysis population and are summarized as mean ± standard deviation (SD) and median (range) for continuous variables and frequency and proportion for categorical variables. Safety data are summarized as the number and percentage of patients experiencing an ADR. For effectiveness data, the number and percentage of patients who experienced CR, CRp and PR and those who did not meet the criteria for CR, CRp and PR (categorized as ‘ineffective’) were calculated. All statistical analyses were performed using SAS software version 9.2–9.4 (SAS Institute Inc., Cary, NC, USA).

## Results

### Patients

The patient disposition is shown in Supplementary Fig. S1. In the all-case survey, 264 patients from 115 study sites were enrolled, and 262 CRFs were collected. The safety and effectiveness analysis populations included 260 and 225 patients, respectively. In the monotherapy survey, 31 patients from 24 study sites were enrolled, and 30 CRFs were collected. The safety and effectiveness analysis populations included 27 and 20 patients, respectively. In the combination therapy survey, 88 patients from 44 study sites were enrolled, and 87 CRFs were collected. The safety and effectiveness analysis populations included 87 and 34 patients, respectively.

Among the 262, 30 and 87 patients evaluated in the all-case, monotherapy and combination therapy surveys, 152 (58.0%), 23 (76.7%) and 44 (50.6%) discontinued treatment, respectively (Supplementary Table S1). The most common reasons for discontinuation in all three surveys were insufficient effectiveness and primary disease progression.

The causes of death are summarized in Table 1. Among the 260 patients in the all-case survey (safety analysis population), 121 (46.5%) died. Most deaths were due to the primary disease (81.0%, 98/260), followed by AEs (19.8%, 24/260).

**Table 1:** Causes of death

	All-case survey *n* = 260	Monotherapy *n* = 27	Combination therapy *n* = 87
Death			
No	139 (53.5)	15 (55.6)	59 (67.8)
Yes	121 (46.5)	12 (44.4)	28 (32.2)
Primary disease	98 (81.0)	12 (100.0)	22 (78.6)
Adverse event	24 (19.8)	1 (8.3)	6 (21.4)
Other	5 (4.1)	0 (0.0)	2 (7.1)

Data are *n* (%).

The background demographic and clinical characteristics of the study populations are summarized in Table 2. In the all-case survey (*N* = 260), 157 (60.4%) of the patients were male, and the mean ± SD and median (range) age were 22.5 ± 19.0 years and 16.0 (0–73) years, respectively. The reason for undergoing treatment with clofarabine was relapsed/refractory ALL in 266 (86.9%) patients. The number of patients with hepatic complications and renal dysfunction was 33 (12.7%) and 10 (3.9%), respectively. Most patients (162 [62.3%] patients) were treated with clofarabine for one cycle; two cycles, 65 (25.0%) patients; three cycles, 20 (7.7%) patients; four cycles, 7 (2.7%) patients; five cycles, 2 (0.77%) patients and six cycles, 4 (1.5%) patients. In the monotherapy and combination therapy surveys, the mean ± SD age was 33.7 ± 20.3 and 11.8 ± 10.3 years, respectively. The background demographic and clinical characteristics of patients aged ≤21 years are summarized in Supplementary Table S2.

**Table 2:** Background patient characteristics (safety analysis population)

	All-case survey *n* = 260		Monotherapy *n* = 27		Combination therapy *n* = 87	
Age, years						
Mean ± SD	22.5 ± 19.0		33.7 ± 20.3		11.8 ± 10.3	
Median (range)	16.0 (0–73)		25.0 (5–66)		10.5 (0–67)	
<15	124	(47.7)	6	(22.2)	67	(77.0)
≥15	133	(51.2)	21	(77.8)	19	(21.8)
Unknown	3	(1.2)	0	(0.0)	1	(1.2)
Sex, male	157	(60.4)	16	(59.3)	49	(56.3)
Diagnosis (reason for using clofarabine)						
Relapsed/refractory acute lymphoblastic leukaemia	226	(86.9)	25	(92.6)	85	(97.7)
Other	34	(13.1)	2	(7.4)	2	(2.3)
Complications						
Hepatic	33	(12.7)	4	(14.8)	5	(5.8)
Renal	10	(3.9)	3	(11.1)	1	(1.2)
Combination therapy with antitumour drugs cyclophosphamide and etoposide (including any dose/other concomitant medications)	116	(44.6)	0	(0.0)	84	(96.6)
Combination therapy with antitumour drugs other than cyclophosphamide and etoposide	47	(18.1)	1	(3.7)^a^	1	(1.2)
Monotherapy with clofarabine (no other concomitant antitumour drugs)^b^	97	(37.3)	26	(96.3)	2	(2.3)
Status at the disease onset: French-American-British classification^c^						
L1	125	(55.3)	8	(32.0)	66	(77.7)
L2	70	(31.0)	14	(56.0)	13	(15.3)
L3	9	(4.0)	0	(0.0)	4	(4.7)
Other	4	(1.8)	1	(4.0)	0	(0.0)
Unknown	9	(4.0)	1	(4.0)	1	(1.2)
Missing	9	(4.0)	1	(4.0)	1	(1.2)
Status at the disease onset: type of leukaemia cells						
B cell	184	(81.4)	17	(68.0)	75	(88.2)
T cell	34	(15.0)	7	(28.0)	7	(8.2)
Other	8	(3.5)	1	(4.0)	3	(3.5)
Number of relapses						
None	23	(10.2)	1	(4.0)	2	(2.4)
1	111	(49.1)	12	(48.0)	52	(61.2)
2	65	(28.8)	10	(40.0)	25	(29.4)
3	22	(9.7)	0	(0.0)	6	(7.1)
≥4	5	(2.2)	2	(8.0)	0	(0.0)
Performance status immediately before clofarabine administration						
0	94	(41.6)	9	(36.0)	42	(49.4)
1	73	(32.3)	5	(20.0)	25	(29.4)
≥2	58	(25.7)	11	(44.0)	18	(21.2)
Missing	1	(0.4)	0	(0.0)	0	(0.0)

Data are *n* (%) unless otherwise specified.

^a^After enrollment, it was revealed that this patient had not received monotherapy.

^b^Any drugs other than antitumour drugs may be included.

^c^This includes patients whose reason for using clofarabine was having a diagnosis of relapsed/refractory acute lymphoblastic leukaemia (all-case survey, *n* = 226; monotherapy, *n* = 25; combination therapy, *n* = 85).

SD, standard deviation.

### Safety

The ADRs and serious ADRs are summarized in Table 3. In the all-case survey, ADRs were observed in 217 of the 260 patients (83.5%). The most common ADRs were hematologic toxicities and included platelet count decrease in 42.7% of patients (*n* = 111); anaemia, 33.9% (*n* = 88); white blood cell count decrease, 33.1% (*n* = 86); neutrophil count decrease, 26.5% (*n* = 69); febrile neutropenia, 25.4% (*n* = 66); bone marrow failure and neutropenia, 5% (*n* = 13) each; haemoglobin decrease, 2.7% (*n* = 7); lymphocyte count decrease, 2.7% (*n* = 7); thrombocytopenia and pancytopenia, 2.3% (*n* = 6) and leukopenia and hematotoxicity, 1.9% (*n* = 5) each. The incidences of ADRs related to infectious disease were as follows: sepsis in 15 patients (5.8%), pneumonia in 7 (2.7%), herpes zoster and infection in 6 (2.3%) each and device-related infection in 3 (1.2%). Regarding hepatobiliary disorders, hepatic function abnormal was reported in 23 patients (8.9%) and liver disorder in 10 (3.9%). Regarding renal disorders, acute kidney injury was reported in five (1.9%) patients and renal impairment in three (1.2%). Tumour lysis syndrome was reported in 10 patients (3.9%); SIRS in 8 (3.1%); capillary leak syndrome in 6 (2.3%) and pericardial infusion in 3 (1.2%).

**Table 3:** Adverse events, adverse drug reactions and serious adverse drug reactions

	All-case survey						Monotherapy						Combination therapy					
Adverse events of special interest/Preferred term	Adverse events		Adverse drug reactions		Serious adverse drug reactions		Adverse events		Adverse drug reactions		Serious adverse drug reactions		Adverse events		Adverse drug reactions		Serious adverse drug reactions	
	*n*	(%)	*n*	(%)	*n*	(%)	*n*	(%)	*n*	(%)	*n*	(%)	*n*	(%)	*n*	(%)	*n*	(%)
Total number of patients	260						27						87					
Hematotoxicity	199	(76.5)	185	(71.2)	144	(55.4)	19	(70.4)	17	(63.0)	15	(55.6)	72	(82.8)	67	(77.0)	52	(59.8)
Platelet count decreased	117	(45.0)	111	(42.7)	70	(26.9)	7	(25.9)	6	(22.2)	5	(18.5)	45	(51.7)	44	(50.6)	29	(33.3)
Anaemia	91	(35.0)	88	(33.9)	43	(16.5)	3	(11.1)	3	(11.1)	3	(11.1)	38	(43.7)	36	(41.4)	16	(18.4)
White blood cell count decreased	87	(33.5)	86	(33.1)	59	(22.7)	5	(18.5)	5	(18.5)	5	(18.5)	33	(37.9)	32	(36.8)	18	(20.7)
Febrile neutropenia	74	(28.5)	66	(25.4)	52	(20.0)	5	(18.5)	5	(18.5)	5	(18.5)	34	(39.1)	31	(35.6)	24	(27.6)
Neutrophil count decreased	73	(28.1)	69	(26.5)	47	(18.1)	7	(25.9)	6	(22.2)	6	(22.2)	30	(34.5)	29	(33.3)	16	(18.4)
Bone marrow failure	13	(5.0)	13	(5.0)	13	(5.0)							10	(11.5)	10	(11.5)	10	(11.5)
Neutropenia	14	(5.4)	13	(5.0)	9	(3.5)	3	(11.1)	3	(11.1)	1	(3.7)	2	(2.3)	1	(1.2)	1	(1.2)
Haemoglobin decreased	8	(3.1)	7	(2.7)	4	(1.5)	2	(7.4)	1	(3.7)	1	(3.7)	2	(2.3)	2	(2.3)		
Lymphocyte count decreased	7	(2.7)	7	(2.7)	5	(1.9)	2	(7.4)	2	(7.4)	2	(7.4)	4	(4.6)	4	(4.6)	1	(1.2)
Thrombocytopenia	7	(2.7)	6	(2.3)	4	(1.5)							2	(2.3)	1	(1.2)	1	(1.2)
Cytopenia	7	(2.7)	3	(1.2)	3	(1.2)												
Disseminated intravascular coagulation	6	(2.3)	2	(0.8)	1	(0.4)	1	(3.7)	1	(3.7)			2	(2.3)	1	(1.2)	1	(1.2)
Pancytopenia	6	(2.3)	6	(2.3)	4	(1.5)	1	(3.7)	1	(3.70)	1	(3.7)	1	(1.2)	1	(1.2)		
Leukopenia	5	(1.9)	5	(1.9)	3	(1.2)	2	(7.4)	2	(7.4)	2	(7.4)	1	(1.2)	1	(1.2)		
Hematotoxicity	5	(1.9)	5	(1.9)	3	(1.2)	1	(3.7)	1	(3.7)			2	(2.3)	2	(2.3)	2	(2.3)
Thrombotic microangiopathy	2	(0.8)					1	(3.7)					1	(1.2)				
Histiocytosis hematophagic	2	(0.8)	1	(0.4)	1	(0.4)												
Lymphocyte count increased	1	(0.4)																
Lymphocytopenia	1	(0.4)	1	(0.4)	1	(0.4)							1	(1.2)	1	(1.2)	1	(1.2)
Infections	70	(26.9)	50	(19.2)	40	(15.4)	6	(22.2)	2	(7.4)	2	(7.4)	26	(29.9)	22	(25.3)	18	(20.7)
Sepsis	23	(8.9)	15	(5.8)	13	(5.0)	1	(3.7)					8	(9.2)	7	(8.1)	6	(6.9)
Pneumonia	10	(3.9)	7	(2.7)	6	(2.3)							2	(2.3)	2	(2.3)	1	(1.2)
Herpes zoster	6	(2.3)	6	(2.3)	4	(1.5)							2	(2.3)	2	(2.3)	2	(2.3)
Infection	6	(2.3)	6	(2.3)	4	(1.5)							2	(2.3)	2	(2.3)	2	(2.3)
Cellulitis	4	(1.5)	2	(0.8)	1	(0.4)	1	(3.7)					1	(1.2)	1	(1.2)		
Bronchopulmonary aspergillosis	3	(1.2)	1	(0.4)	1	(0.4)							2	(2.3)	1	(1.2)	1	(1.2)
Sepsis shock	3	(1.2)	1	(0.4)	1	(0.4)	1	(3.7)	1	(3.7)	1	(3.7)	1	(1.2)	1	(1.2)	1	(1.2)
Device related infection	3	(1.2)	3	(1.2)	1	(0.4)							2	(2.3)	2	(2.3)	1	(1.2)
Pneumonia cytomegaloviral	2	(0.8)	1	(0.4)	1	(0.4)							1	(1.2)				
Bacteremia	2	(0.8)	2	(0.8)	1	(0.4)							3	(3.5)	2	(2.3)	2	(2.3)
Pulmonary mycosis	2	(0.8)	1	(0.4)														
Streptococcal sepsis	2	(0.8)	2	(0.8)	2	(0.8)							1	(1.2)	1	(1.2)	1	(1.2)
Cystitis viral	2	(0.8)	2	(0.8)	2	(0.8)												
Cytomegalovirus infection	1	(0.4)	1	(0.4)	1	(0.4)							1	(1.2)	1	(1.2)	1	(1.2)
Endotoxic shock	1	(0.4)	1	(0.4)	1	(0.4)							1	(1.2)	1	(1.2)	1	(1.2)
Escherichia sepsis	1	(0.4)	1	(0.4)	1	(0.4)												
Fungal infection	1	(0.4)	1	(0.4)	1	(0.4)							2	(2.3)	1	(1.2)	1	(1.2)
Human herpesvirus 6 infection	1	(0.4)	1	(0.4)	1	(0.4)							1	(1.2)	1	(1.2)	1	(1.2)
Influenza	1	(0.4)																
Otitis externa	1	(0.4)					1	(3.7)										
Pneumonia adenoviral	1	(0.4)	1	(0.4)	1	(0.4)												
Pneumonia respiratory syncytial viral	1	(0.4)											1	(1.2)				
Upper respiratory tract infection	1	(0.4)	1	(0.4)									1	(1.2)	1	(1.2)		
Varicella	1	(0.4)	1	(0.4)	1	(0.4)												
Systemic mycosis	1	(0.4)	1	(0.4)									1	(1.2)	1	(1.2)		
Klebsiella sepsis	1	(0.4)	1	(0.4)	1	(0.4)							1	(1.2)	1	(1.2)	1	(1.2)
Staphylococcal sepsis	1	(0.4)	1	(0.4)	1	(0.4)							1	(1.2)	1	(1.2)	1	(1.2)
Adenoviral hemorrhagic cystitis	1	(0.4)	1	(0.4)	1	(0.4)												
Staphylococcal infection	1	(0.4)					1	(3.7)										
Enteritis infectious	1	(0.4)																
Cytomegalovirus viraemia	1	(0.4)	1	(0.4)	1	(0.4)												
Viral hemorrhagic cystitis	1	(0.4)	1	(0.4)	1	(0.4)	1	(3.7)	1	(3.7)	1	(3.7)						
Bacterial infection	1	(0.4)	1	(0.4)	1	(0.4)							1	(1.2)	1	(1.2)	1	(1.2)
Pleural infection	1	(0.4)	1	(0.4)	1	(0.4)												
Respiratory syncytial virus infection	1	(0.4)	1	(0.4)	1	(0.4)												
Biliary tract infection	1	(0.4)																
Aspergillus infection	1	(0.4)	1	(0.4)	1	(0.4)												
Varicella zoster pneumonia	1	(0.4)	1	(0.4)	1	(0.4)												
Vascular device infection	1	(0.4)											1	(1.2)				
Renal disorders	19	(7.3)	9	(3.5)	7	(2.7)	3	(11.1)	1	(3.7)	1	(3.7)	5	(5.8)	1	(1.2)	1	(1.2)
Acute kidney injury	9	(3.5)	5	(1.9)	4	(1.5)	1	(3.7)					2	(2.3)	1	(1.2)	1	(1.12)
Renal impairment	6	(2.3)	3	(1.2)	2	(0.8)	2	(7.4)	1	(3.7)	1	(3.7)	1	(1.2)				
Renal disorder	2	(0.8)											1	(1.2)				
Renal failure	2	(0.8)	1	(0.4)	1	(0.4)							1	(1.2)				
Renal tubular disorder	1	(0.4)	1	(0.4)	1	(0.4)												
Hepatobiliary disorders	42	(16.2)	38	(14.6)	23	(8.9)	7	(25.9)	6	(22.2)	2	(7.4)	9	(10.3)	7	(8.1)	5	(5.8)
Hepatic function abnormal	24	(9.2)	23	(8.9)	14	(5.4)	4	(14.8)	4	(14.8)	1	(3.7)	3	(3.5)	3	(3.5)	3	(3.5)
Liver disorder	11	(4.2)	10	(3.9)	5	(1.9)	2	(7.4)	2	(7.4)	1	(3.7)	4	(4.6)	3	(3.5)	1	(1.2)
Hyperbilirubinemia	2	(0.8)	1	(0.4)									1	(1.2)				
Veno-occlusive liver disease	2	(0.8)	1	(0.4)	1	(0.4)	1	(3.7)										
Hepatic failure	1	(0.4)					1	(3.7)										
Hepatic pain	1	(0.4)	1	(0.4)														
Jaundice	1	(0.4)	1	(0.4)	1	(0.4)												
Hepatobiliary disease	1	(0.4)	1	(0.4)	1	(0.4)							1	(1.2)	1	(1.2)	1	(1.2)
Drug-induced liver injury	1	(0.4)	1	(0.4)	1	(0.4)												
Capillary leak syndrome	7	(2.7)	6	(2.3)	4	(1.5)	1	(3.7)	1	(3.7)	1	(3.7)	1	(1.2)				
Capillary leak syndrome	7	(2.7)	6	(2.3)	4	(1.5)	1	(3.7)	1	(3.7)	1	(3.7)	1	(1.2)				
Systemic inflammatory response syndrome	9	(3.5)	8	(3.1)	7	(2.7)	1	(3.7)	1	(3.7)	1	(3.7)						
Systemic inflammatory response syndrome	9	(3.5)	8	(3.1)	7	(2.7)	1	(3.7)	1	(3.7)	1	(3.7)						
Tumour lysis syndrome	10	(3.9)	10	(3.9)	7	(2.7)							1	(1.2)	1	(1.2)	1	(1.2)
Tumour lysis syndrome	10	(3.9)	10	(3.9)	7	(2.7)							1	(1.2)	1	(1.2)	1	(1.2)
Cardiovascular events	10	(3.9)	4	(1.5)	4	(1.5)	2	(7.4)	1	(3.7)	1	(3.7)	3	(3.5)	1	(1.2)	1	(1.2)
Pericardial effusion	4	(1.5)	3	(1.2)	3	(1.2)	1	(3.7)	1	(3.7)	1	(3.7)	1	(1.2)	1	(1.2)	1	(1.2)
Cardiac failure	3	(1.2)					1	(3.7)					1	(1.2)				
Cardiac failure congestive	1	(0.4)											1	(1.2)				
Electrocardiogram QT prolonged	1	(0.4)	1	(0.4)	1	(0.4)												
Tachycardia	1	(0.4)																

Serious ADRs were reported in 170 of the 260 patients (65.4%). The most common serious ADRs related to hematologic toxicity were platelet count decrease, 26.9% (*n* = 70); white blood cell count decrease, 22.7% (*n* = 59); febrile neutropenia, 20.0% (*n* = 52); neutrophil count decrease, 18.1% (*n* = 47); anaemia, 16.5% (*n* = 43); bone marrow failure, 5.0% (*n* = 13); neutropenia, 3.5% (*n* = 9) and lymphocyte count decrease, 1.9% (*n* = 5) each. Serious ADRs related to infection included sepsis in 13 patients (5.0%) and pneumonia in 6 (2.3%,) and those pertaining to hepatobiliary disorders included hepatic function abnormal in 14 (5.4%) and liver disorder in 5 (1.9%) patients. Other serious ADRs included SIRS and tumour lysis syndrome in seven (2.7%) patients each.

In the monotherapy survey, ADRs were observed in 19 of the 27 patients (70.4%). The most common ADRs observed were hematologic toxicities, including neutrophil count decrease and platelet count decrease, 22.2% (*n* = 6) each; and febrile neutropenia and white blood cell count decrease, 18.5% (*n* = 5) each. Among the hepatobiliary disorders, the incidence of hepatic function abnormal was 14.8% (*n* = 4). Serious ADRs were observed in 16 of the 27 patients (59.3%). The most common serious ADRs observed were neutrophil count decrease, 22.2% (*n* = 6); febrile neutropenia, platelet count decrease and white blood cell count decrease, 18.5% (*n* = 5) each and anaemia, 11.1% (*n* = 3).

In the combination therapy survey, ADRs were observed in 73 of the 87 patients (83.9%). The most common ADRs observed were hematologic toxicities and included platelet count decrease, 50.6% (*n* = 44); anaemia, 41.4% (*n* = 36); white blood cell count decrease, 36.8% (*n* = 32); febrile neutropenia, 35.6% (*n* = 31) and neutrophil count decrease, 33.3% (*n* = 29). Serious ADRs were observed in 57 of the 87 patients (65.5%). The most common serious ADRs observed were platelet count decrease, 33.3% (*n* = 29); febrile neutropenia, 27.6% (*n* = 24); white blood cell count decrease, 20.7% (*n* = 18); anaemia and neutrophil count decrease 18.4% (*n* = 16) each; and bone marrow failure, 11.5% (*n* = 10).

### Effectiveness

In the all-case survey, of the 225 patients in the effectiveness analysis population, 139 were patients with relapsed/refractory ALL aged younger than 22 years. After excluding the one non-evaluable case, among the 138 patients evaluated, 29.7% (*n* = 41) had CR, 7.3% (*n* = 10) had CRp, 10.9% (*n* = 15) had PR and 52.2% (*n* = 72) were classified as ineffective. The CR + CRp rate (remission rate) was 37.0% (*n* = 51), and the CR + CRp + PR rate (effectiveness rate) was 47.8% (*n* = 66). The best overall response rates in the population aged ≥22 years are shown in Supplementary Table S3.

In the monotherapy survey, of the 20 patients in the effectiveness analysis population, 5.0% (*n* = 1) had CR, none (*n* = 0) had CRp, 5.0% (*n* = 1) had PR and 90.0% (*n* = 18) were classified as ineffective. The CR + CRp rate (remission rate) and CR + CRp + PR rate (effectiveness rate) were 5.0% (*n* = 1) and 10.0% (*n* = 2), respectively.

In the combination therapy survey, of the 34 patients in the effectiveness analysis population, 35.3% (*n* = 12) had CR, 8.8% (*n* = 3) had CRp,14.7% (*n* = 5) had PR and 41.2% (*n* = 14) were classified as ineffective. The CR + CRp rate (remission rate) and CR + CRp + PR rate (effectiveness rate) were 44.1% (*n* = 15) and 58.8% (*n* = 20), respectively.

The results of tumour response in all the patients (regardless of age) in the all-case, monotherapy and combination therapy surveys are summarized in Table 4. Effectiveness was confirmed after more than one cycle of clofarabine was administered.

**Table 4:** Tumour response (effectiveness analysis population)

	All-case survey							Monotherapy							Combination therapy						
	*N*	*n*	CR + CRp		CR + CRp + PR		Ineffective	*N*	*n*	CR + CRp		CR + CRp + PR		Ineffective	*N*	*n*	CR + CRp		CR + CRp + PR		Ineffective
			*n* (%)	95% CI	*n* (%)	95% CI	*n* (%)			*n* (%)	95% CI	*n* (%)	95% CI	*n* (%)			*n* (%)	95% CI	*n* (%)	95% CI	*n* (%)
No. of cycles																					
1	134	134	19 (14.2)	8.76–21.25	32 (23.9)	16.94–32.01	102 (76.1)	13	13	1 (7.7)	0.19–36.03	2 (15.4)	1.92–45.45	11 (84.6)	17	17	2 (11.8)	1.46–36.44	5 (29.4)	10.31–55.96	12 (70.6)
2	63	63	33 (52.4)	39.41–65.12	43 (68.3)	55.31–79.42	20 (31.8)	5	5	0 (0.0)	0.00–45.07	0 (0.0)	0.00–45.07	5 (100)	14	14	11 (78.6)	49.20–95.34	13 (92.9)	66.13–99.82	1 (7.1)
≥3	28	27	10 (37.0)		14 (51.9)		13 (48.2)	2	2	0 (0.0)		0 (0.0)		2 (100)	3	3	2 (66.7)		2 (66.7)		1 (33.3)
Total	225	224	62 (27.7)	21.93–34.03	89 (39.7)	33.28–46.46	135 (60.3)	20	20	1 (5.0)	0.13–24.87	2 (10.0)	1.23–31.70	18 (90.0)	34	34	15 (44.1)	27.19–62.11	20 (58.8)	40.70–75.35	14 (41.2)

*N*, number of patients; *n*, number of analysed patients.

CI, confidence interval; CR, complete remission; CRp, complete remission without platelet recovery; PR, partial remission

## Discussion

This study evaluated the safety and effectiveness of clofarabine in Japanese patients with relapsed/refractory ALL in real-world clinical practice. This study is important because to date, evidence of the real-world safety and effectiveness of clofarabine in Japanese patients with relapsed/refractory ALL has been limited.

Regarding the safety findings, in the all-case survey, ADRs were observed in 83.5% of the patients. The most common ADRs were hematotoxicity-related events, similar to ADRs reported in previous clinical trials (13–16).

The effectiveness rate (CR + CRp + PR rate) was 47.8% in the patients with relapsed/refractory ALL aged ≤21 years from the all-case survey, 10.0% in the monotherapy survey and 58.8% in the combination therapy survey. Of note, the effectiveness rate of clofarabine monotherapy was notably lower than the effectiveness rate in previous studies of paediatric patients with relapsed/refractory ALL who were treated with clofarabine monotherapy (30%) (11) and combination therapy (56–64%) (17,18). This trend showing less effectiveness with monotherapy in this study may be related to differences between the studies regarding patient age (unlike previous studies, which included only paediatric patients, the present monotherapy survey included both paediatric and adult patients) and other background characteristics. In addition, the number of patients included in the monotherapy survey (*n* = 27) was very limited. The discontinuation rate in the monotherapy survey was higher than that in the combination therapy survey, as was the proportion of patients with ‘Other AEs (including worsening of complications)’. Discontinuations occurred more frequently in the first cycle in the monotherapy survey than in the combination therapy survey. In a recent Phase 3 study of clofarabine added to intensive treatment in adult patients with newly diagnosed ALL, the addition of clofarabine failed to improve outcomes in this patient population (19). This result in adult patients with ALL, along with the observation that variations in the prevalence of chromosomal alterations, which can affect treatment responsiveness, exist between patients of different ages (20), may partly explain the relatively low effectiveness shown in this study. Thus, the risk and benefit of treatment with clofarabine in adult patients with ALL should be carefully considered. Furthermore, in a previous study of paediatric patients with multiple relapsed or refractory ALL who received clofarabine in combination with cyclophosphamide and etoposide, the probability of overall survival was significantly higher in patients with B-lineage vs T-lineage (33% vs 0%; *P* < 0.001) (18). The immunophenotype may have affected the results in the real world as well.

This study has some limitations, including those inherent to post-marketing surveys (e.g. non-interventional, non-controlled, observational study design). Additionally, the sample size of the monotherapy survey was very small, and the generalizability of the study findings is limited to the Japanese population.

In conclusion, this study provides real-world evidence of the safety and effectiveness of clofarabine administered as monotherapy and in combination with etoposide and cyclophosphamide in Japanese patients with relapsed/refractory ALL. These results indicate that clofarabine monotherapy was effective in Japanese patients, but its effectiveness was considerably lower than with combination therapy. No new safety concerns were raised.

## Supplementary Material

Supplementary_Table_S1_hyae047

Supplementary_Table_S2_hyae047

Supplementary_Table_S3_hyae047

Supplementary_Figure_S1_hyae047

## Data Availability

This post-marketing surveillance was conducted under the Japanese Ministerial Ordinance on Good Post-marketing Study Practice for Drugs, and because of the characteristics of the surveillance in the regulation, the scope of permission for data sharing is limited to the content described in the paper; however, any inquiries should be directed to the corresponding author.
