# Supplementary material for: Safety and effectiveness of clofarabine in Japanese patients with relapsed/refractory acute lymphoblastic leukaemia: a post-marketing surveillance study
Source: Jpn J Clin Oncol. 2024 Apr 20;54(7):778–86. doi: 10.1093/jjco/hyae047 (PMC11228829; doi:10.1093/jjco/hyae047)
Supplement: Supplementary_Table_S1_hyae047 [file supplementary_table_s1_hyae047.docx]

**SUPPLEMENTARY MATERIALS**

**Supplementary Table S1.** Discontinuation

|  | All-case survey  *n* = 262 | | Monotherapy  *n* = 30 | | Combination therapy  *n* = 87 | |
| --- | --- | --- | --- | --- | --- | --- |
| Completed^a^ | 110 | (42.0) | 7 | (23.3) | 43 | (49.4) |
| Discontinued | 152 | (58.0) | 23 | (76.7) | 44 | (50.6) |
| Reason for discontinuation |  |  |  |  |  |  |
| Insufficient effectiveness | 54 | (35.5) | 6 | (26.1) | 20 | (45.5) |
| Primary disease progression | 51 | (33.6) | 8 | (34.8) | 17 | (38.6) |
| Death | 22 | (14.5) | 3 | (13.0) | 5 | (11.4) |
| Other adverse events (including worsening of complications) | 17 | (11.2) | 3 | (13.0) | 3 | (6.8) |
| Other | 17 | (11.2) | 3 | (13.0) | 3 | (6.8) |
| Timing of discontinuation |  |  |  |  |  |  |
| 1st cycle | 108 | (41.2) | 17 | (56.7) | 28 | (32.2) |
| 2nd cycle | 26 | (9.9) | 4 | (13.3) | 9 | (10.3) |
| 3rd cycle | 11 | (4.2) | 1 | (3.3) | 4 | (4.6) |
| 4th cycle | 4 | (1.5) | 1 | (3.3) | 2 | (2.3) |
| 5th cycle | 1 | (0.4) | 0 | (0.0) | 0 | (0.0) |
| 6th cycle | 2 | (0.8) | 0 | (0.0) | 1 | (1.2) |

Data are *n* (%).

^a^Complete remission achieved or treatment (regimen) completed
