# Supplementary material for: Safety and effectiveness of clofarabine in Japanese patients with relapsed/refractory acute lymphoblastic leukaemia: a post-marketing surveillance study
Source: Jpn J Clin Oncol. 2024 Apr 20;54(7):778–86. doi: 10.1093/jjco/hyae047 (PMC11228829; doi:10.1093/jjco/hyae047)
Supplement: Supplementary_Table_S2_hyae047 [file supplementary_table_s2_hyae047.docx]

**Supplementary Table S2.** Background patient characteristics of patients aged ≤21 years

|  | All-case survey | | Monotherapy | | Combination therapy | |
| --- | --- | --- | --- | --- | --- | --- |
|  | Safety analysis  *n* = 161 | Efficacy analysis  *n* = 139 | Safety analysis  *n* = 8 | Efficacy analysis  *n* = 4 | Safety analysis  *n* = 78 | Efficacy analysis  *n* = 33 |
| Age, years |  |  |  |  |  |  |
| Mean ± SD | 10.2 ± 5.7 | 10.4 ± 5.7 | 11.5 ± 5.5 | 10.0 ± 6.3 | 9.3 ± 4.9 | 8.9 ± 4.4 |
| Median (range) | 11.0 (0–21) | 11.0 (0–21) | 12.0 (5–19) | 8.5 (5–18) | 10.0 (0–21) | 9 (1–18) |
| Sex, male | 95 (59.0) | 83 (59.7) | 3 (37.5) | 1 (25.0) | 45 (57.7) | 21 (63.6) |
| Diagnosis (reason for using clofarabine) |  |  |  |  |  |  |
| Relapsed/refractory acute lymphoblastic leukemia | 140 (87.0) | 139 (100.0) | 7 (87.5) | 4 (100.0) | 76 (97.4) | 33 (100.0) |
| Other | 21 (13.0) | 0 (0) | 1 (12.5) | 0 (0.0) | 2 (2.6) | 0 (0.0) |
| Complications |  |  |  |  |  |  |
| Hepatic | 13 (8.1) | 12 (8.6) | 1 (12.5) | 0 (0.0) | 4 (5.1) | 3 (9.1) |
| Renal | 3 (1.9) | 3 (2.2) | 1 (12.5) | 1 (25.0) | 1 (1.3) | 0.(0.0) |
| Heart disease | 5 (3.1) | 5 (3.6) | 0 (0.0) | 0 (0.0) | 3 (3.9) | 1.(3.0) |
| Infectious disease | 12 (7.5) | 10 (7.2) | 1 (12.5) | 0 (0.0) | 6 (7.7) | 4 (12.1) |
| Combination therapy with antitumor drugs cyclophosphamide and etoposide (including any dose/other concomitant medications) | 99 (61.5) | 94 (67.6) | 0 (0.0) | 0 (0.0) | 75 (96.2) | 33 (100.0) |
| Combination therapy with antitumor drugs other than cyclophosphamide and etoposide | 29 (18.0) | 15 (10.8) | 1 (12.5) | 0 (0.0) | 1 (1.3) | 0 (0.0) |
| Monotherapy with clofarabine (no other concomitant antitumor drugs)^a^ | 33 (20.5) | 30 (21.6) | 7 (87.5) | 4 (100.0) | 2 (2.6) | 0 (0.0) |
| Status at the disease onset:  French-American-British classification^b^ |  |  |  |  |  |  |
| L1 | 97 (69.3) | 96 (69.1) | 5 (71.4) | 3 (75.0) | 61 (80.3) | 29 (87.9) |
| L2 | 25 (17.9) | 25 (18.0) | 2 (28.6) | 1 (25.0) | 9 (11.8) | 3 (9.1) |
| L3 | 7 (5.0) | 7 (5.0) | 0 (0.0) | 0 (0.0) | 4 (5.3) | 1 (3.0) |
| Other | 2 (1.4) | 2 (1.4) | 0 (0.0) | 0 (0.0) | 0 (0.0) | 0 (0.0) |
| Unknown | 6 (4.3) | 6 (4.3) | 0 (0.0) | 0 (0.0) | 1 (1.3) | 0 (0.0) |
| Missing | 3 (2.1) | 3 (2.2) | 0 (0.0) | 0 (0.0) | 1 (1.3) | 0 (0.0) |
| Status at the disease onset: type of leukemia cells |  |  |  |  |  |  |
| B cell | 122 (87.1) | 121 (87.1) | 6 (85.7) | 4 (100.0) | 67 (88.2) | 29 (87.9) |
| T cell | 12 (8.6) | 12 (8.6) | 0 (0.0) | 0 (0.0) | 6 (7.9) | 3 (9.1) |
| Other | 6 (4.3) | 6 (4.3) | 1 (14.3) | 0 (0.0) | 3 (4.0) | 1 (3.0) |
| Number of relapses |  |  |  |  |  |  |
| None | 13 (9.3) | 13 (9.4) | 0 (0.0) | 0 (0.0) | 2 (2.6) | 0 (0.0) |
| 1 | 71 (50.7) | 71 (51.1) | 3 (42.9) | 2 (50.0) | 46 (60.5) | 19 (57.6) |
| 2 | 40 (28.6) | 39 (28.1) | 3 (42.9) | 1 (25.0) | 24 (31.6) | 13 (39.4) |
| 3 | 14 (10.0) | 14 (10.1) | 0 (0.0) | 0 (0.0) | 4 (5.3) | 1 (3.0) |
| ≥4 | 2 (1.4) | 2 (1.4) | 1 (14.3) | 1 (25.0) | 0 (0.0) | 0 (0.0) |
| Performance status immediately before clofarabine administration |  |  |  |  |  |  |
| 0 | 65 (46.4) | 65 (46.8) | 4 (57.1) | 3 (75.0) | 40 0 (52.6) | 15 (45.5) |
| 1 | 42 (30.0) | 42 (30.2) | 1 (14.3) | 1 (25.0) | 21 (27.6) | 9 (27.3) |
| 2 | 15 (10.7) | 15 (10.8) | 1 (14.3) | 0 (0.0) | 3 (4.0) | 2 (6.1) |
| 3 | 13 (9.3) | 12 (8.6) | 0 (0.0) | 0 (0.0) | 10 (13.2) | 6 (18.2) |
| 4 | 4 (2.9) | 4 (2.9) | 1 (14.3) | 0 (0.0) | 2 (2.6) | 1 (3.0) |
| Missing | 1 (0.7) | 1 (0.7) | 0 (0.0) | 0 (0.0) | 0 (0.0) | 0 (0.0) |

Data are *n* (%) unless otherwise specified.

^a^Any drugs other than antitumor drugs may be included.

^b^This includes patients whose reason for using clofarabine was having a diagnosis of relapsed/refractory acute lymphoblastic leukemia (all-case survey: safety analysis set, *n* = 140 and efficacy analysis set, *n* = 139; monotherapy: *n* = 7 and 4, respectively; combination therapy: *n* = 76 and 33, respectively).

SD, standard deviation
