# Supplementary material for: Safety and effectiveness of clofarabine in Japanese patients with relapsed/refractory acute lymphoblastic leukaemia: a post-marketing surveillance study
Source: Jpn J Clin Oncol. 2024 Apr 20;54(7):778–86. doi: 10.1093/jjco/hyae047 (PMC11228829; doi:10.1093/jjco/hyae047)
Supplement: Supplementary_Table_S3_hyae047 [file supplementary_table_s3_hyae047.docx]

**Supplementary Table S3.** Best overall response in the population aged ≥22 years (effectiveness analysis population, *n* = 84)

|  | CR | CRp | PR | CR+CRp+PR | Ineffective |
| --- | --- | --- | --- | --- | --- |
| *n* | 4 | 6 | 12 | 22 | 62 |
| % | 4.8 | 7.1 | 14.3 | 26.2 | 73.8 |

CR, complete remission; CRp, CR without platelet recovery; PR, partial remission
