# Supplementary figures and images for: Safety and effectiveness of clofarabine in Japanese patients with relapsed/refractory acute lymphoblastic leukaemia: a post-marketing surveillance study
Source: Jpn J Clin Oncol. 2024 Apr 20;54(7):778–86. doi: 10.1093/jjco/hyae047 (PMC11228829; doi:10.1093/jjco/hyae047)

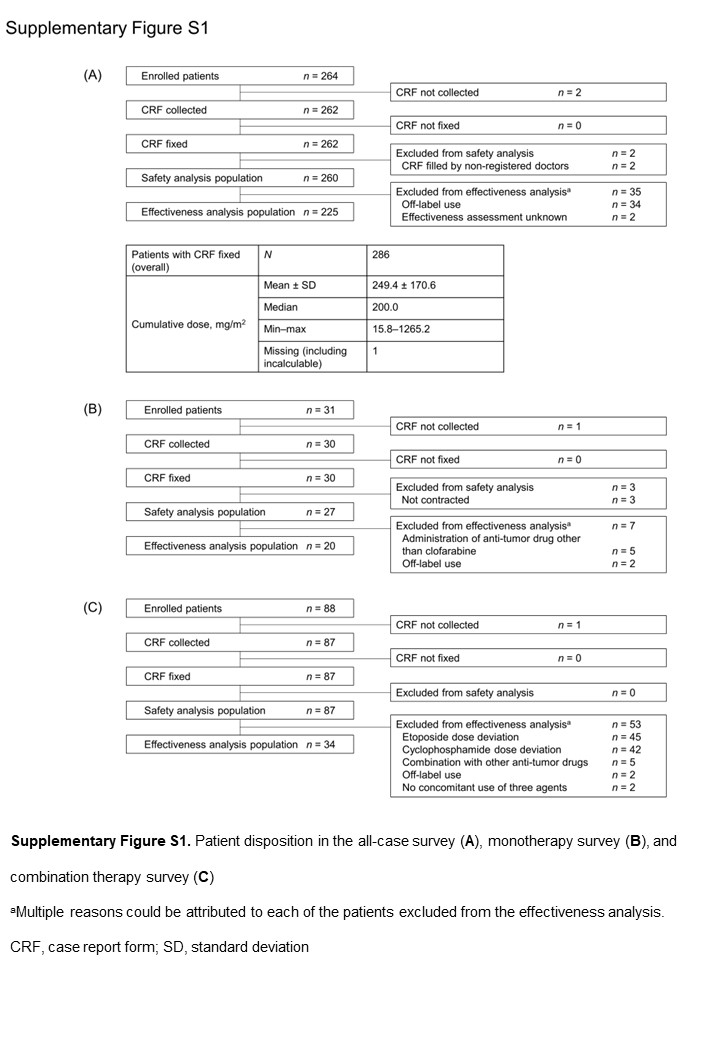

Supplement: Supplementary_Figure_S1_hyae047 [file supplementary_figure_s1_hyae047.jpeg]
